# Potential Use of Alginate-Based Carriers As Antifungal Delivery System

**DOI:** 10.3389/fmicb.2017.00097

**Published:** 2017-01-30

**Authors:** Cristina de Castro Spadari, Luciana B. Lopes, Kelly Ishida

**Affiliations:** ^1^Departamento de Microbiologia, Instituto de Ciências Biomédicas, Universidade de São PauloSão Paulo, Brazil; ^2^Departamento de Farmacologia, Instituto de Ciências Biomédicas, Universidade de São PauloSão Paulo, Brazil

**Keywords:** antifungal, alginate, drug delivery systems, nanoparticles, amphotericin B

## Abstract

Fungal infections have become a major public health problem, growing in number and severity in recent decades due to an increase of immunocompromised patients. The use of therapeutic agents available to treat these fungal infections is limited by their toxicity, low bioavailability, antifungal resistance, and high cost of treatment. Thus, it becomes extremely important to search for new therapeutic options. The use of polymeric systems as drug carriers has emerged as a promising alternative to conventional formulations for antifungals. Alginate is a natural polymer that has been explored in the last decade for development of drug delivery systems due to its non-toxicity, biodegradability, biocompatibility, low cost, mucoadhesive, and non-immunogenic properties. Several antifungal agents have been incorporated in alginate-based delivery systems, including micro and nanoparticles, with great success, displaying promising *in vitro* and *in vivo* results for antifungal activities, reduction in the toxicity and the total drug dose used in the treatment, and improved bioavailability. This review aims at discussing the potential use and benefits of alginate-based nanocarriers and other delivery systems containing antifungal agents in the therapy of fungal infections.

## Introduction

Fungal infections have become a major public health problem and are growing in number and severity over the past three decades. The development of new medical treatments, including therapy with immunosuppressive agents and chemotherapy of cancer, led to a dramatic increase in the number of immunocompromised individuals who are vulnerable to infections which otherwise would have been easily resolved (Karkowska-Kuleta et al., 2009). More than 1.7 billion people worldwide are estimated to suffer from fungal diseases, ranging from superficial to invasive infections (Brown et al., 2012), and some of these infections cause more deaths per year than tuberculosis or malaria (Gaffi – Global Action Fund for Fungal Infections, 2016). However, deaths resulting from invasive or chronic fungal infections are often overlooked, and most public health agencies present little or no mycological surveillance programs (Brown et al., 2012). Preventive measures, premature diagnosis, and the availability of appropriate antifungal treatment could reduce death rates by fungal infections.

The current antifungal therapy is based on three major chemical classes: echinocandins, polyenes, and azoles. Echinocandins are β(1,3)-glucan synthase inhibitors, disrupting the synthesis of the cell wall β(1,3)-D-glucan polymer. Polyenes and azoles alter cytoplasm membrane permeability; polyenes bind irreversibly to ergosterol whilst azoles inhibit ergosterol biosynthesis specifically inhibiting cytochrome P450 dependent enzyme lanosterol 14-α-demethylase. Other antifungals as allylamines and pyrimidine analogs find use against a few fungal infections, mainly to treat cutaneous mycosis (Ostrosky-Zeichner et al., 2010). It is important to emphasize that most antifungal compounds are approved for treatment of superficial mycoses, especially in topical formulations (Denning and Bromley, 2015). In contrast, the clinical repertoire for treatment of invasive fungal infections (IFIs) is much smaller, with only nine approved compounds: amphotericin B, fluconazole, voriconazole, itraconazole, posaconazole, caspofungin, anidulafungin, micafungin, and flucytosine (Denning and Bromley, 2015).

However, the use of these compounds for IFIs is complicated by their poor solubility and bioavailability due to problems in drug absorption and distribution. The situation is aggravated by difficulties to deliver the drug to its target, which leads to an increased incidence of adverse effects resultant from the lack of drug selectivity, which compromise efficacy and safety. An example is the polyene amphotericin B; the drug displays a broad spectrum of action and fungicidal effect, but the significant number of serious adverse effects (which include nephrotoxicity) limits its wider therapeutic use (Hamill, 2013). Triazole agents also exhibit adverse effects associated mainly to hepatic disorders, and their interaction with other drugs have been described due to their ability to modulate the activity of cytochrome P450 enzymes and the hepatic oxidative metabolism of many drugs (Peyton et al., 2015). Even the newest antifungal agents, the echinocandins, present low oral bioavailability and cause gastrointestinal effects after intravenous administration (Chen et al., 2011).

This scenario becomes more complicated if we take into consideration the challenges related to the development of new antifungal agents. The fact that fungi are evolutionarily close to their human host impairs the discovery of new compounds with selective action (Seneviratne and Rosa, 2016). These factors associated with increases in antifungal resistance (Pfaller, 2012), emergence of uncommon fungi (Davoudi et al., 2015; Roilides, 2016), and high mortality rates (Brown et al., 2012; Gaffi – Global Action Fund for Fungal Infections, 2016) have led the scientific community to search for other ways to improve drug targeting and efficacy while reducing adverse effects.

**FIGURE 1:**
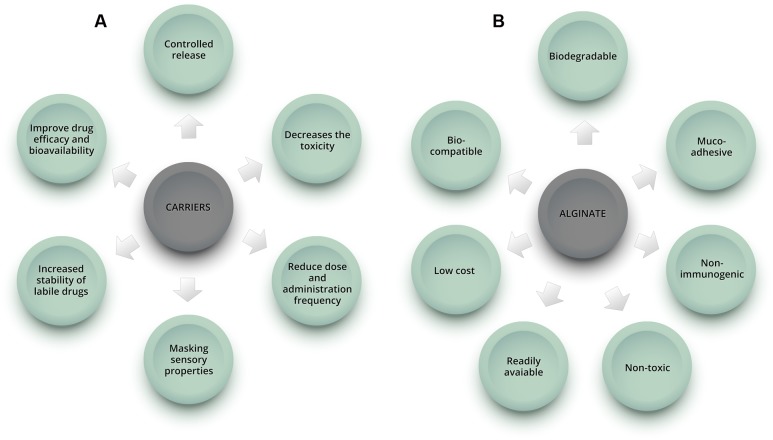
**Schematic representation showing some of the benefits of using carriers in drug formulations** **(A)** and advantages of alginate natural polymer **(B)**.

In this context, drug delivery systems emerge as promising alternatives to improve therapy and overcome the above-mentioned limitations (**Figure 1A**). They have been demonstrated to reduce toxicity caused by conventional drug treatments, improve drug efficacy and bioavailability, reduce dose and administration frequency, and might allow oral administration of compounds with unfavorable pharmacokinetic characteristics, formerly used in topical and/or intravenous forms (Kumar et al., 2013). Various nanocarriers, such as dendrimers, polymers, liposomes, nanoemulsions, and micelles have been investigated for drug delivery (Jeevanandam et al., 2016).

Amphotericin B lipid formulations are examples of the successful application of drug nanocarriers to improve therapeutic outcomes. Three lipid formulations of amphotericin B are available for medical use: liposomal (L-AMB); colloidal dispersion (ABCD); and a lipid complex (ABLC). All were developed to reduce nephrotoxicity without compromising the antifungal efficacy (Falci et al., 2015). The L-AMB formulation (Ambisome^®^) incorporates the drug into liposomes, lipid vesicles of 55–75 nm in diameter composed of soy lecithin, cholesterol, and distearoylphosphatidylglycerol. In the ABCD formulation (Amphocil^®^), amphotericin B is co-incorporated with cholesterol sulfate in microdiscs of an average diameter of 122 nm. The ABLC formulation (Abelcet^®^) is a macromolecular complex of amphotericin B with dimyristoylphosphatidylcholine and dimyristoylphosphatidylglycerol forming ribbon-like particles ranging in length from 1,600 to 6,000 nm (Tiphine et al., 1999; Martinez, 2006; Voltan et al., 2016).

Amphotericin B association with lipids improves drug dissolution, facilitates parenteral infusion, protects the drug from destruction by enzymatic degradation and/or host immune factor inactivation and changes the pharmacokinetic profile of amphotericin B by slow drug release resulting in the protection of potentially vulnerable tissues (most importantly, the kidneys) (Hamill, 2013). In a review, the lipid-based compounds were associated with less risk of changes in renal function, while the risk of a doubling in serum creatinine from baseline was 58% less likely with the lipid based formulations (Barrett et al., 2003). Moreover, the slow and continuous release of amphotericin B from lipid formulations allow the administration of higher doses and longer treatment (Hamill, 2013). However, although lipid-based amphotericin B formulations have shown equivalent efficacy and reduced toxicity compared with the conventional amphotericin B formulation, they are often reserved as a secondary treatment option, presumably because of the differences in costs (Wade et al., 2013).

Various other antifungal agents could benefit from the ability of delivery systems to modify pharmacokinetics and reduce adverse effects. In fact, several studies have been conducted on incorporation of other antifungal agents in various types of delivery systems in the past years, suggesting a demand for novel cost-effective and safe delivery platforms. In this review, we will discuss non-lipid drug carriers for antifungal agents, emphasizing the relevance of alginate-based carriers as an interesting approach for delivery of antifungal agents.

## Polymeric Systems

Among the many types of modified release systems developed, polymer-based systems have attracted attention as they offer significant advantages over other carrier platforms primarily due to the tremendous versatility of polymer matrices, which allows for tailoring of the carrier properties to meet the specific intended need. Other advantages of polymeric systems include ease of production, high encapsulation efficiency of the molecule of interest, protection of the drug against physicochemical degradation, flexibility of their physicochemical properties (such as size, surface charge, and hydrophobicity), slow or fast polymer degradation and stimuli-responsive polymer erosion for temporal control over the release of drugs (Van Vlerken et al., 2007; Voltan et al., 2016).

Bulk gels, films, micro and nanocarriers have been prepared using natural polymers, such as alginate (Liakos et al., 2013; Elsayed et al., 2015), chitosan (Al-Kassas et al., 2016; Islam and Ferro, 2016), gelatin (Mahor et al., 2016), dextran (Gallovic et al., 2016), or synthetic polymers, such as polylactic acid (PLA) (Tyler et al., 2016), poly(lactide-co-glycolide) (PLGA) (Han et al., 2016; Thomas et al., 2016) and others. Natural polysaccharides are advantageous compared to the synthetic polymers due to their abundance in nature, low processing cost, biocompatibility, biodegradability, water solubility, bioactivity, and environmental safety (Sundar et al., 2010; Debele et al., 2016). Among the biopolymers used, alginate represents an excellent alternative for obtainment of drug carriers and is the main focus of this review.

## Alginate

**FIGURE 2:**
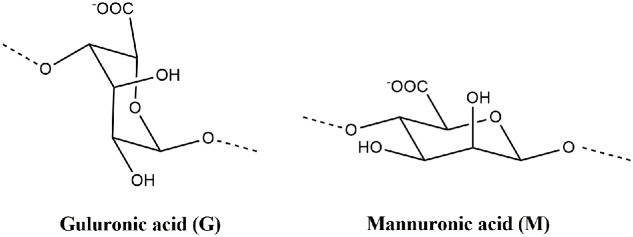
**Molecular structures of α-L-guluronic acid (G) and β-D-mannuronic acid (M) from alginate polymer**.

Alginate is a generic name assigned to a series of natural unbranched polyanionic polysaccharides of β-D-mannuronic acid (M) and α-L-guluronic acid (G) (**Figure 2**) repeating units linked by a 1→4 linkage and displaying chain homosequences of MMM and GGG, interdispersed with MGM heterosequences (Lee and Mooney, 2012; Sosnik, 2014). Molecular weights in the range of 32–400 kg/mol together with different relative G/M compositions and variations in the proportion and chain arrangements of M and G blocks might be found depending on type of seaweed from which alginate was extracted (Sosnik, 2014; Cardoso et al., 2016).

This polysaccharide is frequently obtained from brown seaweeds, including *Laminaria hyperborea*, *Laminaria digitata*, *Laminaria japonica*, *Ascophyllum nodosum*, and *Macrocystis pyrifera* (Lee and Mooney, 2012). Alginate extraction is conducted by treatment with aqueous alkali solutions, typically sodium hydroxide, in which the natural alginate in various salt forms is converted into water-soluble sodium alginate; the extract is filtered, and either sodium or calcium chloride is added to the filtrate in order to precipitate alginate. After further purification and conversion, water-soluble sodium alginate powder is produced (Qin, 2008; Lee and Mooney, 2012). On a dry weight basis, the alginate contents are 22–30% for *A. nodosum*, 25–44% for *L. digitata*, and 17–33 and 25–30%, respectively, for the fronds and stems of *L. hyperborea* (Qin, 2008).

Another alginate source is bacteria from the *Azotobacter* and *Pseudomonas* species. The alginate biosynthesis pathway in bacteria can be divided into four different stages: (i) synthesis of precursor substrate, (ii) polymerization and cytoplasmic membrane transfer, (iii) periplasmic transfer and modification, and (iv) export through the outer membrane. However, even under optimized fermentation conditions, production yields are quite low (around 4 g/l) (Remminghorst and Rehm, 2006), and the process is not considered economically viable for commercial applications (Goh et al., 2012).

Alginate has important characteristics that make it one of the most used polymers for drug delivery application: it is non-toxic, biocompatible, non-immunogenic, biodegradable, mucoadhesive, readily available and has low cost (**Figure 1B**) (Hosseini et al., 2013; Paques et al., 2014). Various modified drug delivery systems can be obtained using alginate, such as hydrogels (polymeric networks with three-dimensional configuration capable of imbibing high amounts of water or biological fluid), microparticles (diameter from 1 to 200 μm) and nanoparticles (diameter from 10 to 1000 nm) (Hamidi et al., 2008; Sosnik, 2014). Depending on the desired application and formulation characteristics, different techniques can be used to produce the carriers. The ionotropic gelation is the most common method used to obtain alginate-based nano and microparticles, beads and hydrogels, among other systems, due to its simplicity and non-toxic procedure.

In this process, alginate is crosslinked in aqueous solutions ionotropically by a mechanism whereby pendant carboxylic acid moieties of G units chelate Ca^2+^ or other divalent cations (e.g., Sr^2+^, Ba^2+^) to generate 3D networks that allow the incorporation of compounds (Tønnesen and Karlsen, 2002; Sosnik, 2014). This gelation is known as the “egg box model” and has been demonstrated to result from specific and strong interactions between calcium ions and blocks of guluronic acid residues (Braccini and Pérez, 2001). Biomolecules and other drugs can be loaded under mild conditions and retained in the 3D structure (Paques et al., 2014; Lopes et al., 2016). Ionotropic gelation can be divided into external and internal gelation. The main difference between these methods is the type of calcium salt used and consequently the gelling kinetics (Draget et al., 2005).

In the external gelation method, which is also referred to as the “diffusion method” particles are produced by dropping a drug-loaded alginate solution into the aqueous solution of a soluble salt of calcium, such as calcium chloride. The internal gelation method, also referred to as “internal setting,” is characterized by the release of calcium ions in a controlled manner from an insoluble calcium source, such as calcium carbonate, within the alginate solution. Controlled release of calcium is usually obtained by a change in pH, by a limited solubility of the calcium salt source, and/or by the presence of chelating agents (Draget et al., 2005; Paques et al., 2014; Lopes et al., 2016).

Micro and nanoparticles of alginate are usually obtained using emulsification (W/O or O/W) followed by external or internal gelation (Hosseini et al., 2013; Paques et al., 2013; Song et al., 2013). Emulsions are basically obtained by dispersing a fluid into another immiscible fluid and are produced by fragmenting, through various processes, one phase into another, and leading to a dispersion of droplets in a continuous phase. The recombination of the freshly formed droplets may be partially suppressed by the addition of surfactants that locate at the droplet interface reducing the interfacial tension (Bibette and Leal-Calderon, 1996; Pillai et al., 1999).

## Alginate-Based Delivery Systems for Antifungal Drugs

Several types of alginate-based delivery systems for antifungal agents have been studied in the last few years, such as nanoparticles and microparticles, beads, hydrogels, tablets, and films. In the next sections, alginate–based carriers for two classes of antifungal agents will be addressed: azoles and polyenes (**Table 1**).

**Table 1:** Alginate-based drug delivery systems for antifungal agents azoles and polyenes.

Antifungal	Carrier	Therapeutic goal	Experimental model	Reference
Clotrimazole	Alginate nanoparticles	Improve bioavailability of the drug	*In vivo* (mice)	Pandey et al., 2005
	
	Films of sodium alginate, hydroxyl propylcellulose and propylene glycol	Treatment of vaginal candidiasis	*In vitro*	Mishra et al., 2016

Econazole	Alginate nanoparticles	Improve bioavailability of the drug	*In vivo* (mice)	Pandey et al., 2005
	Alginate nanoparticles	Chemotherapeutic potential against murine tuberculosis	*In vivo* (mice)	Ahmad et al., 2007

Miconazole	Tablets of alginate	Evaluation of the buccal bioadhesive properties of slow-release tablets containing miconazole	*In vitro* and *in vivo* (human voluntary)	Mohammed and Khedr, 2003

Fluconazole	Films of sodium alginate and polyvinyl alcohol	Overcome the problems of high drug dose requirement and toxicity	*In vitro* and *ex vivo*	Patel et al., 2015
	Ethyl cellulose microspheres with internal phase of alginate	Antifungal activity against *Candida albicans*	*In vitro*	Maiti et al., 2009
	Alginate mucoadhesive films and disks	Topical treatment of oral candidiasis	*In vitro* and *in vivo* (human voluntary)	Yehia et al., 2008, 2009

Voriconazole	Pluronic and sodium alginate gels	Ocular drug delivery against *C. albicans* and *Aspergillus fumigatus*	*In vitro*	Pawar and Edgar, 2012

Amphotericin B	Sodium alginate nanospheres	Treatment of systemic candidiasis	*In vitro* and *in vivo* (mice)	Sangeetha et al., 2007
	Sodium alginate-glycol chitosan stearate nanoparticles	for better chemotherapy of visceral leishmaniasis	*In vitro* and *in vivo* (hamster)	Gupta et al., 2015
	Alginate films with starch Pickering emulsions	Treatment of oral candidiasis	*In vitro*	Cossu et al., 2015
	Alginate-capped amphotericin B lipid	Treatment of visceral leishmaniasis	*In vitro*	Singodia et al., 2011

Nystatin	Alginate beads, micro- and nanoparticles incorporated in toothpaste	Increase the effectiveness of the nystatin in the treatment of oral candidiasis	*In vitro*	Reis et al., 2015
	Alginate microspheres and alginate hydrogel	Treatment of oral candidiasis	*In vitro* and *in vivo* (pigs)	Martín et al., 2015
	Alginate microparticles, chitosan and poloxamer 407 coated alginate microparticles	Treatment of *Candida* vaginitis	*In vitro* and *ex vivo*	Martín-Villena et al., 2013


### Azoles

Azoles inhibit the fungal cytochrome P450 (CYP) enzyme 14-α-sterol-demethylase, which is involved in ergosterol biosynthesis, a major sterol component of fungal cytoplasmic membranes (Kathiravan et al., 2012). Therapeutic use of azoles displays two main problems. The first one is their ability to interact with mammalian P450 isozymes, affecting the metabolism of other drugs that serve as substrates of these enzymes, which may result in significant drug interactions including reduced efficacy or increased incidence of adverse effects. The second drawback relates to the low aqueous solubility of several azoles: miconazole, ketoconazole, itraconazole, and posaconazole are all slightly water-soluble (<1 μg/ml) or insoluble at neutral pH (Yang et al., 2008). Low aqueous solubility is known to severely limit azole bioavailability after oral administration and its therapeutic effectiveness. The low concentration of the drug at the infection site may also induce antifungal resistance to some less susceptible species (Yang et al., 2008). The first limitation is more easily addressed by local drug delivery to the site of infection, which can be prolonged/controlled using alginate-based carriers. Systemic administration of alginate carriers can overcome the second, as they have been demonstrated to improve the bioavailability of antifungal imidazoles and triazoles in various studies, improving efficacy while potentially reducing the frequency of drug administration.

#### Imidazoles

Econazole and clotrimazole have been mainly used topically due to the low absorption after oral administration (Fromtling, 1988). To improve the oral bioavailability, Pandey et al. (2005) encapsulated these antifungals separately in alginate nanoparticles. When free drug was administered, it was detected in the plasma of animals for up to 3–4 h. In contrast, after administration of the alginate nanoparticles, econazole, and clotrimazole could be detected for longer periods of time (5–6 days). In addition, the drugs were detected in the lung, liver, and spleen up to 8 days following administration of the nanoparticles, while free antifungals were cleared within 12 h. Thus, reduced frequency of antifungal administration was proposed as a potential advantage of the alginate nanoparticles containing econazole or clotrimazole.

Ahmad et al. (2007) evaluated the chemotherapeutic potential of alginate nanoparticles encapsulating econazole and antitubercular drugs (ATDs) against murine tuberculosis. Azole drugs have proven their antimycobacterial potential under *in vitro*, *ex vivo*, and *in vivo* conditions against murine tuberculosis caused by susceptible, resistant, and latent bacilli (Ahmad et al., 2006). They observed that co-encapsulation of these drugs in alginate nanoparticles reduced the dosing frequency of econazole and ATDs by 15-fold, suggesting the advantage of their encapsulation for sustained release of these drugs (Ahmad et al., 2007).

Other studies demonstrated the applicability of alginate to obtain mucoadhesive carriers for treatment of mucosal candidiasis. Mishra et al. (2016) investigated mucoadhesive films of clotrimazole for vaginal administration, a patient-convenient alternative for treatment of vaginal candidiasis. Conventional vaginal dosage forms, such as creams, foams, jellies have short residence time at the site of application, resulting in reduced therapeutic effect. Vaginal films possess several advantages including the ease of storage and handling, no need of an applicator, and better drug stability (Hussain and Ahsan, 2005; Mishra et al., 2016). The optimized film provided higher drug release than the marketed product Candid-V6^®^ vaginal tablet (70% drug release in 1 h and 83% drug release in 6 h while marketed product showed 14% drug release in 1 h and 41% drug release in 6 h). In addition, the films maintained *in vitro* antifungal activity without inhibiting *Lactobacillus* growth (Mishra et al., 2016). Mohammed and Khedr (2003) obtained slow-release buccal bioadhesive alginate tablets of miconazole, and demonstrated that these tablets markedly prolonged the duration of antifungal activity of the drug in the saliva of human volunteers for more than 8 h compared with the commercial miconazole gel (Daktaren oral gel), improving patient convenience.

#### Triazoles

Maiti et al. (2009) prepared ethyl cellulose microspheres with high entrapment levels of fluconazole by alginate facilitated water-in-oil-in-water (w/o/w) emulsion solvent evaporation technique. The drug entrapment efficiency (%) in ethyl cellulose microspheres, prepared by conventional w/o/w emulsion solvent evaporation technique, was found to be 10.93 ± 1.23, while the addition of 2% (w/v) sodium alginate in the internal aqueous phase of the multiple emulsion improved the efficiency of drug entrapment to 80% (Maiti et al., 2009), most likely due to an increase in the viscosity of the internal phase (Gainza et al., 2013). In addition, *in vitro* antifungal activity of fluconazole incorporated in ethyl cellulose-alginate microspheres against *Candida albicans* (MIC ranges from 1.29 to 5.69 μg/ml) were comparable to the MIC values of free fluconazole (0.12 to >64 μg/ml) (Maiti et al., 2009). These studies suggested that the use of aqueous sodium alginate solution as an internal phase in conventional double emulsion solvent evaporation technique may be an alternative approach for the successful incorporation of slightly water soluble drugs in microspheres obtained with ethyl cellulose and other synthetic polymers (Maiti et al., 2009; Gainza et al., 2013).

Mucoadhesive disks and films (Yehia et al., 2008, 2009) containing a small dose of fluconazole were developed for topical treatment of oral candidiasis to ensure satisfactory drug levels in the mouth for prolonged time periods, reducing systemic adverse effects and the possibility of drug–drug interaction. Compared to other polymers (hydroxypropyl methyl cellulose, hydroxyethyl cellulose, chitosan, eudragit), the alginate-based disks containing fluconazole were considered the best formulation in terms of adhesion characteristics, residence times and release rates both in *in vitro* assays and *in vivo* using the oral mucosa of human volunteers (Yehia et al., 2009). The alginate disks containing fluconazole presented swelling and adhesion properties, increased *in vivo* residence time and prolonged drug release over approximately 5 h (Yehia et al., 2008). Recently, Patel et al. (2015) developed bioadhesive films of fluconazole to provide localized drug delivery exclusively at the site of infection, thereby reducing its total dose and hence, dose-related toxicity. The time necessary for complete erosion or detachment of the film was over 5 h in an *ex vivo* model of rat skin surface using a modified IP (Indian Pharmacopeia) disintegration apparatus; and the *in vitro* assays demonstrated a controlled release of the fluconazole up to 8 h using a diffusion cell method (Patel et al., 2015).

Pawar et al. (2013) evaluated the use of a pluronic/alginate-based gel for ophthalmic delivery of voriconazole. All batches of formulations were mucoadhesive and displayed a loading efficiency between 95 and 100%. Importantly, the antifungal efficiency of the formulation against *C. albicans* and *Aspergillus fumigatus in vitro* confirmed that the designed formulation has prolonged effect and retained its antifungal properties against fungal infection. The local voriconazole bioavailability obtained with alginate-based gel ranged from 19.74 to 30.82% in 4 h (Pawar et al., 2013) and it was considered an important advantage to conventional systems, such as eye drops, suspensions, and ointments that result in less than 5% of administered drug entering the eye (Gaudana et al., 2009).

### Polyenes

Polyene antibiotics form a complex with ergosterol and disrupt the fungal plasma membrane, increasing membrane permeability and leakage of the cytoplasmic contents, which results in the ultimate death of the fungal cell (Kathiravan et al., 2012). Polyene macrolides are rather toxic, causing serious side effects, such as renal failure, hypokalemia, and thrombophlebitis, especially upon intravenous administration. Besides that, these drugs are poorly soluble in water, and can easily form aggregates of micelles in aqueous media. This feature is apparently responsible for the difficulties encountered during administration of polyene macrolides including poor absorption after oral administration and poor distribution of the antibiotics in organs and tissues (Zotchev, 2003).

Amphotericin B alginate films containing starch Pickering emulsion were demonstrated to be a good alternative for treatment of mucosa candidiasis. *In vitro* results demonstrated efficacy of the emulsions dispersed in alginate films against *C. albicans* at concentrations above 5 μg/mL (Cossu et al., 2015). The inhibitory effect of amphotericin B in the microbiological assay showed that the addition of 100 U/mL α-amylase resulted in an enhanced effect on *C. albicans* due to the increased bioavailability of amphotericin B released from the emulsions. This result demonstrated that the release of amphotericin B from starch Pickering emulsions in alginate films could be controlled by α-amylase in the system, an interesting feature to provide release of hydrophobic antifungal, such as amphotericin B (Cossu et al., 2015).

Alginate nanospheres containing amphotericin B for treatment of systemic candidiasis in a murine model displayed higher antifungal efficacy in comparison to the conventional formulation of amphotericin B (Sangeetha et al., 2007). In the study, amphotericin B-containing nanospheres administered intravenously for 7 days led to a mice survival rate of 80% (compared to 60% for free drug) as well as 10-fold lower fungal load in the affected organs (liver and lungs) compared with the free drug (Sangeetha et al., 2007). The authors suggested that alginate nanospheres containing amphotericin B might improve biodistribution, enhancing drug localization in the liver and lungs while reducing it in the kidneys. As a result, a reduction in total dose as well as dose-related systemic adverse effects mainly in the kidneys was observed (Sangeetha et al., 2007). Gupta et al. (2015) produced amphotericin B-loaded alginate glycol chitosan stearate nanoparticles that presented efficacy in a visceral leishmaniasis hamster model after intraperitoneal administration. Drug encapsulation in the nanoparticles modified its tissue distribution, with higher drug concentrations being localized in *Leishmania* infected organs (i.e., spleen and liver) and lower concentrations in the kidneys, reducing toxicity compared to the free drug (Gupta et al., 2015).

Another study used amphotericin B in an alginate-capped lipid nanocarrier (Alg-Lip-nano) for leishmaniasis treatment (Singodia et al., 2011). *In vitro* results demonstrated that the percentage of parasite inhibition (intramacrophagic amastigotes) of Alg-Lip-nano (58%) was significantly higher compared to Lip-nano without alginate (48%) (*p* ≤ 0.05). This supports the authors hypothesis that alginate coating over lipid particles activates macrophages to release pro-inflammatory cytokines, which synergistically act with amphotericin B and can potentially increase the cure rate in visceral leishmaniasis (Singodia et al., 2011).

Some work has also been carried out with the polyene agent nystatin. This compound is recommended only for topical administration due to its high toxicity. Martín-Villena et al. (2013) obtained alginate microparticles containing nystatin through the emulsification/internal gelation method with an encapsulation efficiency of 80% for treatment of *Candida* vaginitis. After *ex vivo* permeation studies through porcine vaginal mucosa, determination of the amount of nystatin retained and antifungal activity assays, the authors inferred that the developed microparticulate system was efficacious against *C. albicans* without systemic absorption of toxic concentrations of the drug (Martín-Villena et al., 2013). More specifically, the formulation was able to adhere to the vaginal mucosa showing potential for safe treatment of localized infection. Nystatin release from microcapsules followed a concentration gradient pattern based on the first Fick’s law (release rate constant: 1.60 h^-1^) offering sustained release (Martín-Villena et al., 2013). Moreover, nystatin-loaded microparticles exhibited a clear inhibition effect on the *C. albicans* growth (Martín-Villena et al., 2013).

Alginate microspheres containing nystatin demonstrated ability to control *Candida* infection in the oral cavity of pigs without causing damage to the oral tissue and systemic nystatin absorption (Martín et al., 2015). Another study for treatment of oral candidiasis showed an improved nystatin effectiveness using beads, micro- and nanoparticles of alginate incorporated in toothpaste instead of free drug in suspension form (Reis et al., 2015). This study demonstrated that microparticles were the most suitable particulate system of nystatin showing the slowest release (complete release within 3 h for beads, 24 h for nanoparticles, and 48 h for microparticles), the highest inhibitory effect on *C. albicans* (MIC value of 13.5 μg/mL for beads, 38 μg/mL for nanoparticles, and 5.21 μg/mL for microparticles) and a high antifungal efficiency over time (1 year of evaluation) (Reis et al., 2015).

## Alginate-Based Delivery Systems for Non-Conventional Antifungals

Alginate formulations have been used as delivery systems for other molecules with antifungal activity potential, such as essential oils, antiseptics, and others (**Table 2**).

**Table 2:** Examples of alginate-based delivery systems for non-conventional antifungals.

	Types of materials incorporated	Carrier	Reference
Essential oils	Turmeric	chitosan-alginate nanocapsules	Lertsutthiwong and Rojsitthisak, 2011
	Turmeric	Alginate nanocapsules	Lertsutthiwong et al., 2008
	
	*Satureja hortensi*	Alginate microparticles	Hosseini et al., 2013
	
	Elicriso italic, chamomile blue, cinnamon, lavender, tea tree, peppermint, eucalyptus, lemongrass, and lemon	Alginate films	Liakos et al., 2014
	
	Clove, thyme, and cinnamon	Alginate microspheres	Soliman et al., 2013
	
	Anise	Alginate gel	Gafiţanu et al., 2016

Antiseptics	Chlorhexidine diacetate	Alginate and alginate/chitosan films	Juliano et al., 2008
	
	Povidone iodine	Alginate beads and films	Liakos et al., 2013

Pomegranate extract		Alginate microparticles	Endo et al., 2012

Lactoferrin		Alginate tablets	Kuipers et al., 2002

Silver		Alginate as a stabilizer silver nanoparticles	Kubyshkin et al., 2016
		
		Cellulose/sodium alginate films	Shao et al., 2015


Turmeric oil from the rhizome of *Curcuma longa* is an example of an essential oil encapsulated in alginate-based carriers. Turmeric oil is widely used as a food additive, condiment and household medicine in Southern Asia, and presents several pharmacological properties as an antibacterial, an antifungal, an antioxidant, an antimutagenic, and a repellent (Lertsutthiwong et al., 2008). Lertsutthiwong et al. (2008) encapsulated this oil in alginate nanocapsules, while Lertsutthiwong and Rojsitthisak (2011) produced chitosan-alginate nanocapsules containing turmeric oil. Chitosan-alginate nanocapsules increased the skin permeation of turmeric oil compared with an ethanolic solution (Lertsutthiwong and Rojsitthisak, 2011), representing a new approach for topical delivery and treatment of cutaneous infections (Lertsutthiwong and Rojsitthisak, 2011). These studies also demonstrated that alginate is an effective biopolymer for encapsulation of a volatile essential oil, producing nanocarriers with long-term physical stability (120 days) when stored at 4°C.

Hosseini et al. (2013) encapsulated *Satureja hortensis* essential oil (known for its antimicrobial and antioxidant activity) in alginate microparticles, and concluded that these microparticles could be used for antioxidant and antimicrobial purposes with controlled release properties in the food and nutraceuticals industries. Other essential oils, such as elicriso italic, chamomile blue, cinnamon, lavender, tea tree, peppermint, eucalyptus, lemongrass, and lemon were dispersed in sodium alginate films and presented remarkable antibacterial and antifungal activities (Liakos et al., 2014). These alginate films enriched with essential oils should find multiple applications, including as disposable wound dressings, in food packaging, medical device protection and disinfection, and indoor air quality improvement materials (Liakos et al., 2014).

Soliman et al. (2013) encapsulated clove, thyme and cinnamon oils in alginate microspheres in order to achieve their optimal antifungal activity against two of mycotoxigenic fungi species, *Aspergillus niger* and *Fusarium verticillioides*, and prevent fungal contamination in stored grains. These microspheres showed antifungal activity in addition to increasing the storage time of oil with antifungal activity for up to 8 days, while the free oil lost activity within 2 days. Thus, the main advantage of encapsulation was to reduce the rate of evaporation of these essential oils prolonging antifungal activity (Soliman et al., 2013).

Plant extracts have also been incorporated in alginate-based nanocarriers. The anise crude extracts and active compounds manifest antimicrobial, antioxidant, insecticidal, analgesic, sedative, and convulsive activities (Wang et al., 2011). Anise-based bioadhesive alginate gels for vaginal application showed antibacterial activity, and no effect against *Candida* species was observed (Gafiţanu et al., 2016). Alginate microparticles containing pomegranate extract (*Punica granatum*) with encapsulation efficiency of 81.9% (Endo et al., 2012) displayed antifungal activity against *C. albicans* (minimum inhibitory concentration of 3.9 μg/ml), with the advantage of providing controlled extract release to achieve the desired therapeutic effect against oral fungal infection caused by *C. albicans* (Endo et al., 2012).

Sodium alginate tablets containing lactoferrin, a potential antifungal candidate for candidiasis treatment, displayed release-controlling and mucoadhesive properties (Kuipers et al., 2002). *In vitro* microbiological studies demonstrated that the formulation had the same antifungal properties as the free lactoferrin against several clinical isolates of *C. albicans* and *Candida glabrata* (Kuipers et al., 2002). The tablets (250 mg lactoferrin) on oral mucosa of human volunteers were able to keep the lactoferrin concentration in the saliva for at least 2 h, suggesting that they might improve the therapeutic efficacy of lactoferrin in oral candidiasis treatment (Kuipers et al., 2002).

Research showed that the use of sodium alginate as a stabilizer provided a highly stable solution of silver nanoparticles (nanosilver) that has significant antibacterial and antifungal activity. The nanosilver aqueous solution at concentrations of 0.0005–0.005% with sodium alginate completely inhibited *C. albicans* viability within 24 h (Kubyshkin et al., 2016). Shao et al. (2015) developed silver sulfadiazine-loaded cellulose/sodium alginate films and observed that these films had excellent antibacterial performances on *E. coli* and *S. aureus* and antifungal activity on *C. albicans.* They also showed good biocompatibility and accepted cytotoxicity (less than 20% using 0.1% of silver sulfadiazine). The authors suggested that these films containing silver sulfadiazine could be a potential antimicrobial for use in wound dressings.

Antiseptics have also been incorporated in alginate carriers for controlled release (Juliano et al., 2008; Liakos et al., 2013). Films of sodium alginate or sodium alginate/chitosan were well tolerated, and able to sustain the release of chlorhexidine diacetate, providing active concentrations against *C. albicans* for prolonged periods of time (approximately 3 h) on the buccal mucosa of human volunteers, which is in agreement with results showing *in vitro* mucoadhesion (Juliano et al., 2008). Another antiseptic incorporated in alginate beads and films was povidone iodine (PVPI) (Liakos et al., 2013). Controlled release of PVPI was possible when the composite films and beads were brought into direct contact with aqueous media, maintaining the bactericidal and fungicidal properties. This characteristic is highly desired in clinical applications to avoid toxic doses of PVPI (Liakos et al., 2013). A wide variety of applications of these systems are envisioned, such as wound dressings, hygienic and protective packaging films for medical devices, and polymer beads as water disinfectants (Liakos et al., 2013).

## Concluding Remarks

Alginate is a natural polymer widely used for obtainment of drug delivery systems due to its non-toxicity, biodegradability, biocompatibility, low cost, mucoadhesive, and non-immunogenic properties, in addition to the availability of simple protocols for production of these systems. In spite of the popularity of alginate-based carriers in the drug delivery field, few studies have been conducted with antifungals, mostly dealing with azoles and polyenes. Thus, there is a gap when it comes to the advantages and limitations of alginate-based carriers for other types of antifungal agents. To the best of our knowledge, this review is the first to directly focus on alginate carriers for antifungal drugs. Based on the studies discussed here, alginate-based antifungal delivery systems have shown great potential in the treatment of fungal infections, providing modified release, and decreasing administration frequency, toxicity and the dose necessary for antifungal efficacy. Therefore, the use of alginate-based carriers represents a promising approach for delivery of antifungal agents to improve the treatment protocols for cutaneous and systemic mycosis.

## Author Contributions

CS did the literature review and wrote the manuscript. KI and LL designed and co-wrote the manuscript.

## Conflict of Interest Statement

The authors declare that the research was conducted in the absence of any commercial or financial relationships that could be construed as a potential conflict of interest.
